# 眼部慢性移植物抗宿主病诊治中国专家共识（2023年版）

**DOI:** 10.3760/cma.j.issn.0253-2727.2023.06.002

**Published:** 2023-06

**Authors:** 中华医学会血液学分会造血干细胞应用学组

慢性移植物抗宿主病（chronic graft-versus-host disease，cGVHD）是异基因造血干细胞移植后常见的并发症之一，可累及多个器官，发生率为30％～70％[1]–[2]。其中，眼部cGVHD的发生率为40％～60％[3]–[4]，半数以上患者伴有视力下降，显著影响患者移植后生活质量[5]–[6]。目前，眼部cGVHD缺乏统一的诊疗规范，血液科与眼科医师的协同治疗需要进一步加强。因此，中华医学会血液学分会造血干细胞应用学组组织相关专家编写此共识，旨在规范眼部cGVHD的诊断治疗，达到早诊早治、控制症状、改善视力、提高生活质量的目的。

一、眼部cGVHD的定义及发病机制

眼部cGVHD是一种异基因造血干细胞移植后发生的眼部并发症，主要是由供者T细胞诱发的炎性反应，发病机制尚未完全阐明。眼部cGVHD的发生机制与T细胞浸润引发的多种免疫损伤相关[7]–[9]。主要表现为供者CD4^+^T细胞和活化的CD8^+^T细胞浸润泪腺，形成促炎环境，巨噬细胞、病理性成纤维细胞募集，造成泪腺损伤与导管纤维化，泪液分泌减少[10]–[11]。眼睑炎症和纤维化改变使眼表易于暴露，发生倒睫，睑板腺损伤[12]–[13]；结膜上皮杯状细胞丢失和瘢痕样改变[14]–[15]；也可以导致角膜上皮损伤、内皮细胞减少和基底下神经密度降低[16]–[18]。淋巴细胞与病理性成纤维细胞也可浸润泪点、泪小管、鼻泪管等结构，造成纤维化与梗阻，出现泪液反流与继发感染[19]–[20]。此外，B细胞可通过产生自身抗体、细胞因子和趋化因子以及作为调节细胞参与眼部cGVHD的免疫过程[21]–[24]。眼表损伤和免疫细胞浸润会产生多种细胞因子和趋化因子，形成促炎信号刺激，反复加重眼表损伤[25]。

二、眼部cGVHD的临床表现和诊断分级

（一）眼部cGVHD的临床表现

眼部cGVHD临床表现较复杂，可累及眼部所有结构，主要包括泪腺、睑板腺和眼睑、结膜、角膜、泪道等，也可出现葡萄膜炎和视网膜病变等并发症[26]。眼部cGVHD的临床症状以干眼为主，其他临床症状还包括眼部疼痛、畏光、刺激、烧灼感、异物感、视物模糊、结膜充血、流泪等[27]–[28]。此外，严重的眼部cGVHD还会导致患者角膜溃疡与穿孔等，导致严重的视物模糊和视力下降[29]–[31]。眼部cGVHD的病理改变和临床表现见表1。

**表1:** 眼部慢性移植物抗宿主病的病理改变和临床表现

病变组织	病理改变	临床表现
泪腺	T细胞与巨噬细胞浸润、细胞因子/趋化因子上调、病理成纤维细胞活化、泪腺导管纤维化与梗阻	干眼症
睑板腺与眼睑	T细胞与巨噬细胞浸润、细胞因子/趋化因子上调、睑板腺导管梗阻	睑板腺功能障碍（MGD）、睑缘炎、倒睫、眼睑内翻和外翻瘢痕性改变
结膜	T细胞、巨噬细胞、中性粒细胞浸润、细胞因子/趋化因子上调、结膜上皮下纤维化、杯状细胞丢失	结膜充血、球结膜水肿、假膜形成、角膜结膜炎（KCS）、瘢痕性结膜炎
角膜	T细胞与巨噬细胞浸润、细胞因子/趋化因子上调、角膜上皮细胞损伤与内皮细胞减少、角膜基底下神经密度降低和角膜新生血管形成	点状角膜病、丝状角膜炎、持续性角膜上皮缺损、角膜溃疡/穿孔
泪道	T细胞与巨噬细胞浸润、细胞因子/趋化因子上调、病理成纤维细胞活化、导管纤维化与梗阻	泪点、泪小管或鼻泪管堵塞、泪囊炎、溢泪

（二）眼部cGVHD的诊断和分级

1. 眼科检查：眼科专科检查是眼部cGVHD诊断、评估及观察疗效的重要手段，建议患者移植之前进行眼科专科检查；移植后出现cGVHD表现，建议患者每3～6个月定期进行眼科检查及后续随访[32]–[33]。眼科检查推荐：视力、眼压、眼底检查、Schirmer Ⅰ试验、荧光素染色泪膜破裂时间（FTBUT）、眼表综合分析仪检查（泪河高度、非接触式泪膜破裂时间、脂质层观察、睑板腺及其开口分析、眼红分析、眼表荧光染色）、活体共聚焦显微镜检查（角膜、睑板腺）、结膜印记细胞学检查等[34]–[35]。此外，泪液生物标志物也可用于眼部cGVHD诊断、疗效评估等。已有研究显示，眼部cGVHD患者泪液中白细胞介素6（IL-6）、IL-8、γ干扰素（IFN-γ）、基质金属蛋白酶-9（MMP-9）等表达水平增高，而趋化因子CXCL-10、表皮生长因子（EGF）等表达水平降低[36]–[41]，泪液生物标志物尚处于研究及探索阶段[42]。具体眼科检查项目及其意义见表2。

**表2:** 眼部慢性移植物抗宿主病临床眼科检查项目及意义

检查项目	参考值	异常结果	临床意义
Schirmer Ⅰ 试验	>10 mm	≤10 mm/5 min	泪液分泌减少
荧光素染色泪膜破裂时间	≥10 s	<10 s	泪膜破裂时间缩短，泪膜不稳定
眼表综合分析仪检查			
泪河高度	>0.2 mm	≤0.2 mm	泪液分泌减少
非接触式泪膜破裂时间	>10 s	≤10 s	泪膜破裂时间缩短，泪膜不稳定
脂质层观察		脂质层色彩单一、分布不均匀、厚度异常	泪液脂质层异常，泪膜不稳定
睑板腺及其开口分析		睑板腺缺失、开口堵塞	睑板腺形态改变、功能障碍
眼红分析		结膜轻度至重度充血	结膜存在炎性反应与损伤
眼表荧光素染色		绿色点状着色、糜烂、片状缺损、溃疡	角膜、结膜损伤
活体共聚焦显微镜检查			
角膜		免疫炎症细胞数量增多、神经纤维形态改变和神经密度降低	角膜炎性反应、角膜损伤、角膜神经功能异常
睑板腺		睑板腺腺泡密度降低、直径变短、导管开口堵塞	睑板腺形态改变、睑板腺功能障碍
结膜印迹细胞学检查		结膜杯状细胞密度降低，核质比增大，鳞状上皮化生	结膜损伤

2. 诊断及分级：

（1）眼部cGVHD的诊断和分期标准Ⅰ[1]：诊断标准见表3。根据所需支持性护理的类型以及症状对日常活动（ADL）的影响，将眼部cGVHD的严重程度分为轻、中、重三个等级，分级标准见表4。本标准简单实用，适合用于临床快速诊断和评估。

**表3:** 眼部慢性移植物抗宿主病的诊断标准

诊断标准（①或②符合任意一条即可诊断）
①基础泪液分泌试验（Schirmer Ⅰ试验）平均值≤ 5 mm/5 min；
②基础泪液分泌试验（Schirmer Ⅰ试验）平均值为6~10 mm/5 min，通过裂隙灯检查新发干燥性角结膜炎；
③排除其他原因引起的眼部并发症。

**表4:** 眼部慢性移植物抗宿主病的严重程度分级

严重程度分级	判定标准	对应分值
	无症状	0
轻度干眼	不影响日常生活（每天使用润滑性滴眼液≤3次）	1
中度干眼	部分影响日常生活（每日使用润滑性滴眼液>3次，或使用泪点栓塞），干燥性角结膜炎未引起新的视力受损	2
重度干眼	显著影响日常生活（需要使用专用护目镜减轻痛苦或因为眼部症状无法工作），或因干燥性角结膜炎导致视力受损	3

（2）眼部cGVHD的诊断和分期标准Ⅱ[43]–[45]：主要依据眼表疾病指数（OSDI）、基础泪液分泌试验（Schirmer Ⅰ test, SIT）、角膜荧光素染色（CFS）和结膜充血四个指标并参考国际眼部cGVHD工作组的建议进行综合分析诊断和严重程度分级，详见表5、表6。本评判标准更加精准，建议选择固定的眼科医师评估，可作为临床研究客观评价指标的诊断和分级。

**表5:** 眼部慢性移植物抗宿主病（cGVHD）诊断标准

系统cGVHD症状	不足以诊断	疑诊	确诊
无	0～5分	6~7分	≥8分
有	0～3分	4~5分	≥6分

注 总评分＝基础泪液分泌试验（Schirmer Ⅰ）评分＋角膜荧光染色评分＋眼表疾病指数（OSDI）评分＋结膜充血评分。不存在系统cGVHD症状时，总评分≤5分不足以诊断，6～7分为疑诊，≥8分为确诊；存在系统cGVHD症状时，总评分≤3分不足以诊断，4～5分为疑诊，≥6分为确诊

**表6:** 眼部慢性移植物抗宿主病严重程度分级量表

总评分（分）	Schirmer Ⅰ 试验（mm）	角膜荧光素染色（分）	OSDI评分（分）	结膜充血（分）
0	>15	0	<13	无
1	11～15	<2	13～22	轻/中
2	6～10	2～3	23～33	重
3	≤5	≥4	>33	—

注 总评分＝基础泪液分泌试验（Schirmer Ⅰ 试验）评分＋角膜荧光染色评分＋眼表疾病指数（OSDI）评分＋结膜充血评分。严重程度评判：轻度/中度：5～8分；严重：9～11分

两个诊断和分期标准之间有适度的一致性，但诊断和分期标准Ⅰ不涉及眼部炎症活动和结膜、角膜受累指标，无法精确评估疾病的严重程度。建议：诊断和分期标准Ⅰ用于患者随访评估，诊断和分期标准Ⅱ作为临床研究客观评价指标的诊断和分级[46]。

（三）鉴别诊断

眼部cGVHD临床表现较复杂，需要与干燥综合征的眼部表现、长期用眼过度、睡眠不足等引起的干眼症、眼部感染、药物不良反应等仔细鉴别，及时的专科会诊或多学科（MDT）综合诊治是必要的。

1. 其他原因导致的干眼症：主要表现为眼干涩、疲劳、异物感、眼痛、畏光等，可以通过裂隙灯显微镜下观察患者结膜瘢痕化情况鉴别[47]。

2. 眼部感染：细菌、真菌感染可以通过角膜刮片、病原学培养鉴别诊断；病毒感染通过眼底检查或眼内液检出病毒DNA加以鉴别[48]–[51]。

3. 药物相关性眼病：根据既往用药史及裂隙灯检查等辅助鉴别诊断[52]–[56]。

三、眼部cGVHD的治疗

（一）治疗原则

应与眼科医师密切合作，推荐采用局部治疗和全身治疗相结合的综合治疗方法。

眼部cGVHD治疗主要有四个治疗目标：润滑眼表、控制引流、控制蒸发和减少眼表炎症。而严重、顽固或复杂的病例可能需要根据患者情况选择包括手术治疗在内的综合治疗措施。

（二）全身治疗

根据《慢性移植物抗宿主病（cGVHD）诊断与治疗中国专家共识（2021年版）》的指导意见，轻度眼部cGVHD患者可观察或进行局部治疗，眼部受累2分以上（中、重度）患者或合并≥2个其他器官受累应考虑进行全身治疗[57]。目前，cGVHD全身治疗药物首选糖皮质激素联合或不联合钙调磷酸酶抑制剂。对于糖皮质激素耐药的cGVHD患者，可以采用二线治疗方法，使用糖皮质激素期间也应注意眼科随访和检查[58]。对于眼部cGVHD患者，不同药物的治疗反应率不尽相同，西罗莫司治疗反应率为50％～64％[59]–[61]、芦可替尼为25％～100％[62]–[64]、利妥昔单抗为0～43％[65]–[66]、间充质干细胞（MSC）为29％～55％[67]–[70]。对于重症患者强调局部治疗与全身治疗联合使用[71]–[74]。

（三）局部治疗

1. 人工泪液：人工泪液已被证实是眼部cGVHD干眼症患者的一线治疗方法[75]。建议轻度干眼患者选择黏稠度较低的人工泪液（0.1％玻璃酸钠、0.5％羧甲基纤维素等），中重度干眼患者选择黏稠度较高的人工泪液（0.3％玻璃酸钠、1％羧甲基纤维素等）。

注意：临床医师应熟悉每一种药物的特点，针对患者个体化治疗，选择单用或不同种类人工泪液联合使用；建议：①人工泪液使用次数过于频繁可能会破坏正常泪膜环境，加快泪液蒸发，每日使用次数不宜超过6次[76]；②开封后最多使用4周；③避免选择含有防腐剂（常见成分包括季铵盐类，如苯扎溴铵、有机汞类、脒类、醇类、酯类等5类）和富含磷酸盐的滴眼制剂[77]。

2. 糖皮质激素：糖皮质激素适用于伴有活动性眼部炎症的眼部cGVHD中重度干眼患者[78]–[80]。糖皮质激素滴眼液用法尚不统一，使用原则：低浓度、短疗程，炎性反应控制后缓慢停药。建议每日1～4次，维持2～4周，实际用药频率应视患者眼表炎性反应严重程度决定[81]。此外，睑缘炎性反应较重者推荐应用糖皮质激素眼膏，每日1～2次，持续1～2周[82]。对于角膜上皮缺损、基质变薄或感染的患者禁用局部糖皮质激素。长期使用糖皮质激素可诱发眼压升高、白内障、青光眼或继发眼部感染等不良反应[59]。建议使用糖皮质激素>2周的患者监测眼压、眼底及视野等指标。

3. 钙调磷酸抑制剂：环孢素A（CsA）适用于活动性或有潜在眼表炎症的眼部cGVHD患者[83]–[85]。建议0.05％ CsA每日2次（主要）或0.1％ CsA每日1次，实际用药频率视患者情况决定。如0.05％ CsA每日2次疗效不佳，可加至每日3～4次[86]。常见的不良反应包括眼部刺激症状、结膜充血和眼部瘙痒等[87]。

他克莫司（FK506）适用于重度眼部cGVHD患者[88]。常用的他克莫司眼用制剂包括软膏和滴眼液，常见浓度为0.05％～0.1％，建议每日2次[89]。据报道，他克莫司可有效改善CsA不耐受、糖皮质激素难治性眼部cGVHD患者眼部症状[90]。常见不良反应包括眼部灼热感、异物感、视物模糊（软膏多见），但无眼压升高[91]。

4. 生物类制剂：

（1）血清类制剂：自体血清适用于角膜上皮损伤的中重度患者，禁用于角膜穿孔和各种活动性角膜炎的眼部cGVHD[92]–[94]。自体血清常见体积分数为20％～100％。对于角膜上皮病变严重者，推荐采用自体血清浓度序贯疗法：先用高浓度（50％～100％）治疗，待角膜上皮修复后改为低浓度（20％）维持治疗[95]。自体血清临床使用存在生产成本高、保存困难、污染风险等问题。此外，眼部cGVHD患者有全身感染性疾病或其他不适宜制备自体血清的情况时，脐带血血清、同种异体血清、血小板源性滴眼液或小牛血去蛋白提取物眼用凝胶也有临床治疗眼部cGVHD的报告[96]–[98]。

（2）眼表修复制剂：眼表修复制剂适用于眼部cGVHD伴有角膜上皮缺损、点状角膜病变的患者。常见药物推荐：重组人表皮生长因子衍生物、重组牛碱性成纤维细胞生长因子等，建议每日2～4次或遵药物说明书[81]。注意：细胞因子类制剂需2～8 °C冷藏，避免高温及避光。

（3）细胞及其衍生物：MSC在移植免疫方面有广泛应用前景。近期报道，间充质干细胞外泌体滴眼液（10 µg/50 ml，每日4次，持续2周）可以减轻眼部cGVHD[99]。MSC的使用方式、最佳剂量、最佳时间、最佳疗程仍需临床探索。

5. 手术治疗：

（1）泪小点栓塞术：适用于重度泪腺功能异常的患者[100]。但泪小点栓塞术作用有限，且可能出现瘙痒、溢泪、栓塞部位肉芽肿和泪小管炎、栓子自发脱落等并发症[101]–[102]。对栓塞材料存在变态反应、患有泪囊炎、感染性结膜炎的患者禁止行泪小点栓塞术[103]。

（2）羊膜移植术：羊膜移植（AMT）适用于难治性上皮缺损和角膜溃疡的眼部cGVHD患者[104]。目前，羊膜移植术在多个个案中均有报道，安全性较高，但对视力的改善情况有待进一步观察[105]。对患有感染性角膜炎的眼部cGVHD患者，建议感染消退后再放置羊膜[106]。

（3）角膜缘干细胞和角膜移植术：角膜缘干细胞移植和角膜移植是治疗眼表疾病的重要手段。角膜缘干细胞移植术适用于角膜缘干细胞缺乏症、严重睑球粘连的患者[107]–[108]。角膜移植术适用于出现严重影响视力的角膜穿孔、白斑等患者，但对于眼表条件差的患者，手术效果有限[109]–[110]。

（四）护理及辅助治疗

对于眼部cGVHD患者的门诊与后续随访工作，建议注重健康宣教与心理干预，并告知日常生活护理注意事项与辅助治疗。

1. 健康宣教与心理干预：对移植后患者，建议进行以下眼部健康宣教：①简单讲解眼部cGVHD疾病基本知识，提高患者认知并引起重视；②合理使用电子产品；③建议新鲜果蔬和适当的有氧运动[111]。此外，眼部cGVHD患者出现心理障碍，临床医师应积极进行沟通、疏导，并劝说患者接受心理专科医师的心理治疗[112]。

2. 日常生活护理：眼部cGVHD，尤其伴睑板腺功能障碍者，推荐以下日常眼部护理：睑缘清洁、热敷熏蒸、睑板腺按摩、干眼雾化等可有效改善眼部cGVHD患者的睑板腺分泌功能；家庭热敷建议用热毛巾、热敷眼罩、加热蒸汽眼罩等，温度40～45 °C，保持10～15 min，每日2～3次[113]。对于出现明显畏光症状的眼部cGVHD患者，建议眼部避光。

3. 辅助治疗：常见的辅助治疗包括佩戴湿房镜、绷带软镜、巩膜镜等[114]–[116]。湿房镜可减少泪液蒸发，减轻症状；绷带镜可减少摩擦、保护眼表，短期使用可有效治疗丝状角膜炎、持续性角膜上皮缺损、复发性角膜糜烂等；巩膜镜也可减少眼睑对角结膜的摩擦，保护眼表。

四、总结

眼部作为cGVHD高发的靶器官，显著影响患者的生活质量，在日益重视移植后生活质量的今天尤为重要[117]–[118]。对于眼部cGVHD提倡“早期筛查、早期诊断、早期干预、规范化治疗和随访观察”。目前，眼部cGVHD的诊治缺乏跨学科共同诊疗及个性化治疗方案，现行的治疗方法也缺乏循证级别较高的临床研究数据支持，需要进一步规范管理并有计划地开展相关临床研究。
